# Biocompatibility Analyses of HF-Passivated Magnesium Screws for Guided Bone Regeneration (GBR)

**DOI:** 10.3390/ijms222212567

**Published:** 2021-11-22

**Authors:** Ole Jung, Bernhard Hesse, Sanja Stojanovic, Christian Seim, Timm Weitkamp, Milijana Batinic, Oliver Goerke, Željka Perić Kačarević, Patrick Rider, Stevo Najman, Mike Barbeck

**Affiliations:** 1 Clinic and Policlinic for Dermatology and Venereology, University Medical Center Rostock, 18057 Rostock, Germany; ole.tiberius.jung@googlemail.com; 2 Xploraytion GmbH, 10625 Berlin, Germany; hesse@xploraytion.com (B.H.); seim@xploraytion.com (C.S.); 3 Department of Biology and Human Genetics, Faculty of Medicine, University of Niš, 18108 Niš, Serbia; sanja.genetika.nis@gmail.com (S.S.); stevo.najman@gmail.com (S.N.); 4 Scientific Research Center for Biomedicine, Faculty of Medicine, Department for Cell and Tissue Engineering, University of Niš, 18108 Niš, Serbia; 5 Synchrotron SOLEIL, Gif-sur-Yvette, 91190 Saint-Aubin, France; weitkamp@synchrotron-soleil.fr; 6 Department of Ceramic Materials, Chair of Advanced Ceramic Materials, Institute for Materials Science and Technologies, Technical University of Berlin, 10623 Berlin, Germany; m.batinic@tu-berlin.de (M.B.); o.goerke@tu-berlin.de (O.G.); 7 Department of Anatomy Histology, Embryology, Pathology Anatomy and Pathology Histology, Faculty of Dental Medicine and Health, University of Osijek, 31000 Osijek, Croatia; zeljkapericc@gmail.com; 8 BerlinAnalytix GmbH, 12109 Berlin, Germany; patrick.rider@botiss.com

**Keywords:** guided bone regeneration (GBR), barrier membrane, fixation, screw, pin, biodegradation, magnesium, in vivo, in vitro, histomorphometry, tissue reaction

## Abstract

**Background:** Magnesium (Mg) is one of the most promising materials for human use in surgery due to material characteristics such as its elastic modulus as well as its resorbable and regenerative properties. In this study, HF-coated and uncoated novel bioresorbable magnesium fixation screws for maxillofacial and dental surgical applications were investigated in vitro and in vivo to evaluate the biocompatibility of the HF coating. **Methods:** Mg alloy screws that had either undergone a surface treatment with hydrofluoric-acid (HF) or left untreated were investigated. In vitro investigation included XTT, BrdU and LDH in accordance with the DIN ISO 10993-5/-12. In vivo, the screws were implanted into the tibia of rabbits. After 3 and 6 weeks, degradation, local tissue reactions and bony integration were analyzed histopathologically and histomorphometrically. Additionally, SEM/EDX analysis and synchrotron phase-contrast microtomography (µCT) measurements were conducted. The in vitro analyses revealed that the Mg screws are cytocompatible, with improved results when the surface had been passivated with HF. In vivo, the HF-treated Mg screws implanted showed a reduction in gas formation, slower biodegradation and a better bony integration in comparison to the untreated Mg screws. Histopathologically, the HF-passivated screws induced a layer of macrophages as part of its biodegradation process, whereas the untreated screws caused a slight fibrous tissue reaction. SEM/EDX analysis showed that both screws formed a similar layer of calcium phosphates on their surfaces and were surrounded by bone. Furthermore, the µCT revealed the presence of a metallic core of the screws, a faster absorbing corrosion front and a slow absorbing region of corroded magnesium. **Conclusions:** Overall, the HF-passivated Mg fixation screws showed significantly better biocompatibility in vitro and in vivo compared to the untreated screws.

## 1. Introduction

Insufficient alveolar ridge dimensions represent a common challenge in edentulous jaws, which prevents an immediate placement of dental implants. To overcome this, an augmentation of the ridge is performed. The process of guided bone regeneration (GBR) is established as the standard procedure in the clinical practice for bone augmentation and has been extensively shown to produce excellent outcomes in a range of clinical studies [1,2,3,4,5]. GBR is based on the principle of excluding fast proliferating cells, such as epithelium cells from the augmentation site, while promoting a micromilieu favorable for jawbone regeneration by osteoblasts [6,7]. The central element of this concept is the barrier membrane, which enables the seclusion of the augmentation site [8,9,10]. A variety of resorbable and non-resorbable membranes are available, each coming with different properties determined by their chemical composition or origin [2,11,12,13,14]. In the last decades, the use of resorbable membranes, most often collagen based, have been established in dentistry, as their application does not require a second surgical intervention for their removal. The progression from non-resorbable to resorbable membranes shows that degradable materials in surgery and specifically in dental implantology are becoming the materials of choice for most clinicians, and a positive trend toward completely bioresorbable material systems [9,15,16,17,18].

Other medical devices used in combination with barrier membranes are fixation pins and screws. These are used for membrane fixation to the local jawbone and support both the insertion and fixation of bone substitute material (BSM). Securing the membrane with pins or screws maximizes the stability of the membrane and allows for a significantly enhanced bone formation [19,20]. Due to their stability, non-resorbable titanium-based pins are still the gold standard in dentistry; however, it is usually required to remove the titanium pins during a second surgery. Alternative resorbable pins have been developed to overcome the necessity for pin removal and have been introduced onto the market [19,21,22,23]. For example, biodegradable polymer pins based on polyglycolic acid (PGA), polylactic acid (PLA) and other homopolymers and copolymers have been studied to replace titanium devices [24,25]. Additionally, pins obtained from decellularized human or bovine cortical bone have been described [19]. However, the application of these resorbable pins has been associated with different issues, such as a low biocompatibility (e.g., due to the degradation products) or a lack of mechanical functionality for maintaining a secure fixation [26,27,28].

In the pursuit of developing new biomaterials for dental bone regeneration in the context of GBR and implantology, magnesium (Mg) and its alloys have received considerable attention due to their good biocompatibility and biodegradable properties [17,29,30,31,32,33]. However, clinical applications might be limited if the degradation behavior is uncontrolled, associated with hydrogen gas release and the production of a local alkaline environment, which can have a negative effect on the healing process if it is too pronounced [31,34]. To overcome the aforementioned challenges, different alloys and coatings/surface passivations were developed [35].

The major principles for surface passivation include conversion (e.g., anodization, chemical conversion or ion implantation) as well as deposition coatings (e.g., electro deposition, spin coating, electrophoretic painting) [16,17,18,31,32,33,35,36]. Furthermore, alloy development with passivating elements such as rare earth or other metallic elements are subjects of multiple studies [37,38].

Treatment of biomedical implant with hydrofluoric acid (HF) as part of the conversion coatings has shown to be an effective strategy for surface passivation in several studies. Passivation of the surface has been reported to reduce corrosion rates up to 20 times [39,40,41,42,43,44]. Through this process, hydroxides, oxides and compounds of the general formula “Mg(OH)xF_2_-x” are formed on the surface of Mg-implants [43,44]. The HF passivation technique used in this study has already been analyzed as part of a two-component barrier membrane composed of collagen and a Mg lattice structure [15]. The results revealed that an HF passivation of the Mg lattice membrane surface significantly improved cytocompatibility and significantly reduced gas cavity formation for up to 12 weeks after implantation. These results led to the conclusion that HF is a promising treatment option to control the biodegradation of Mg-based biomaterials. Thus, the question arises if this passivation method is also suitable to adapt the biodegradation behavior of Mg alloy fixation screws for GBR applications. Thus, this novel biomaterial concept based on resorbable pins, which, even in combination with resorbable materials such as collagen membranes or bone substitutes, can change the GBR concept and minimize the number of required surgical interventions combined with a suitable degradation behavior.

The aim of the present preclinical study was the analysis of the cyto- and biocompatibility, biodegradation behavior and bony integration of newly developed Mg alloy screws for membrane fixation that have had their surface treated with HF (NovaMag^®^ fixation screw, botiss biomaterials GmbH, Zossen, Germany). A control group of untreated Mg alloy screws was used for a comparison (botiss biomaterials GmbH, Zossen, Germany). An in vitro study was designed based on ISO 10993-5/-12 (biological evaluation of medical devices: tests for in vitro cytotoxicity/sample preparation and reference materials). This study design was specifically adapted for Mg-based biomaterials, as previously described [16,17]. According to the in vitro study design, proliferation (BrdU), viability (XTT) and cytotoxicity (LDH) assays were performed. An in vivo study was also conducted, whereby the screws were implanted into the tibia of 40 New Zealand rabbits for 3 and 6 weeks. The in vivo analysis included established histological work-up methods as well as previously published histopathological and histomorphometrical procedures [15,45,46,47,48,49,50]. Additionally, an element analysis based on scanning electron microscopy (SEM) combined with energy dispersive X-ray spectroscopy (EDX) was performed to quantitatively and qualitatively detect the element distribution within the implantation beds of the screws. Finally, Synchrotron-based phase contrast micro-CT was used to analyze the corrosion behavior of the Mg screws.

## 2. Results

### 2.1. Cytocompatibility Analysis

**Figure 1:**
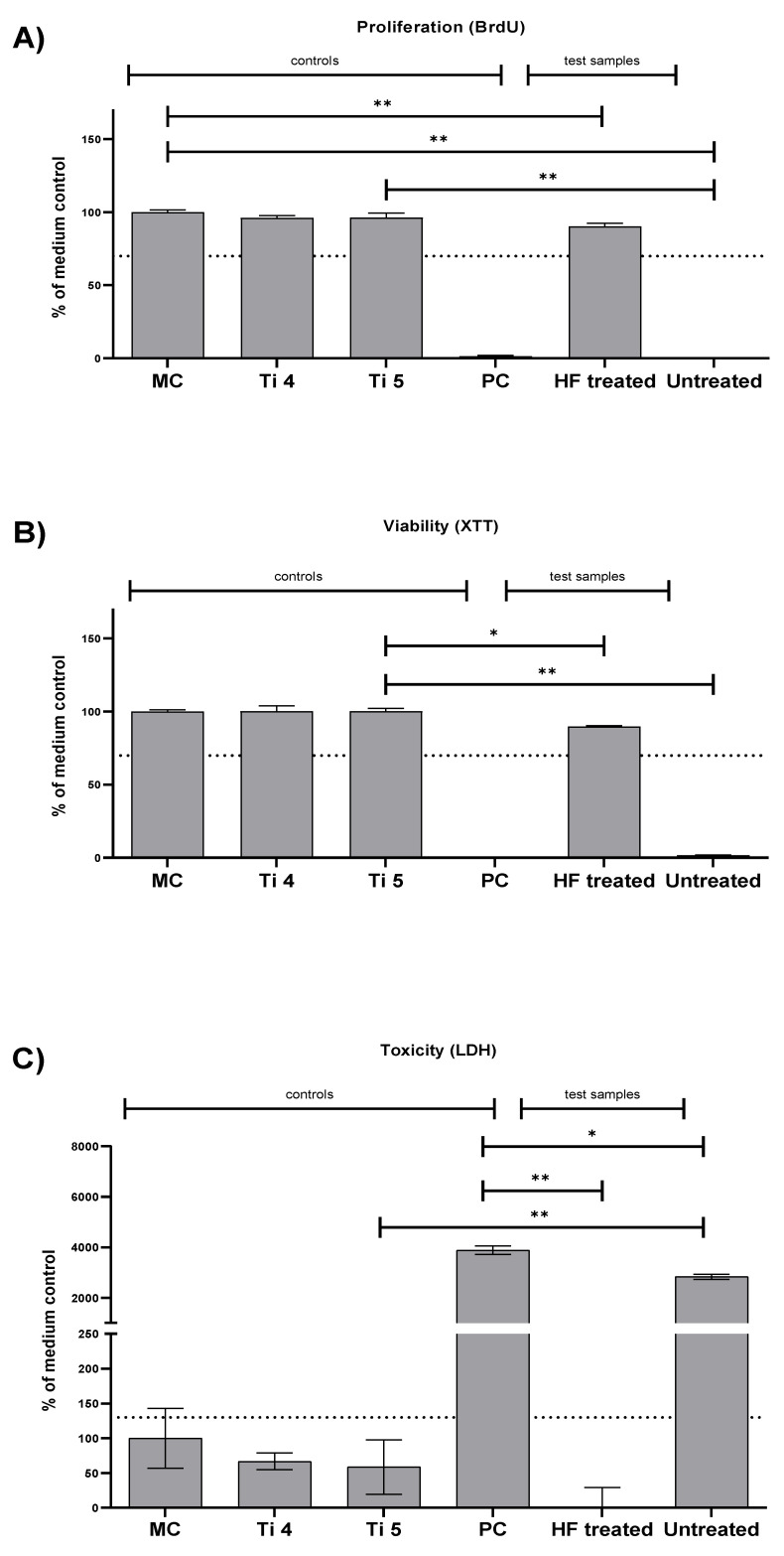
Cytocompatibility results using L929 cells. (**A**) Proliferation measured by the BrdU assay; (**B**) viability measured by the XTT-assay; (**C**) cytotoxicity measured by the LDH assay. Values are compared against either the titanium grade 5 negative control (BrdU, XTT) or the positive control (LDH). Significant differences are marked using asterisks (* *p* ≤ 0.05 and ** *p* ≤ 0.01). Means with error bars indicate standard deviations. The dotted lines indicate thresholds that should not be surpassed (LDH) or undercut (BrdU, XTT). (MC, medium control; Ti 4, titanium grade 4; Ti 5, titanium grade 5; PC, positive control).

ISO 10993-5:2009 defines the nontoxic range for cells cultured with biomaterial extracts as >70% viability of the blank sample (medium control) for BrdU and XTT assays and for values < 130% of the blank sample (medium control) for LDH assays [16,17]. The HF-treated magnesium screws showed suitable cytocompatibility in both the XTT and BrdU assays, whereas the values of both assays for the untreated Mg screws showed less cytocompatibility (Table 1 and Figure 1A,B). Thereby, the HF-treated screws showed only slightly significant differences to the negative reference materials’ titanium grade 5 in the XTT assay and no significant differences in the BrdU assay (Table 1 and Figure 1A,B).

The HF-treated screws demonstrated only a small difference to the titanium grade 5 negative reference material in the XTT assay and no significant difference in the BrdU assay (Table 1 and Figure 1A,B). However, the values of the untreated screws had a substantial significant difference compared to the negative controls (** *p* ≤ 0.01) (Table 1 and Figure 1A,B).

These results are also consistent with that of the LDH assay, where the values of HF-treated screws were below the cytotoxic threshold and significantly different from the positive control (** *p* ≤ 0.01) (Figure 1C). The values in the group of the untreated screws exceeded the threshold and showed significant differences compared to the positive control (* *p* ≤ 0.05) (Figure 1C).

### 2.2. Synchrotron µCT Analysis

**Figure 2:**
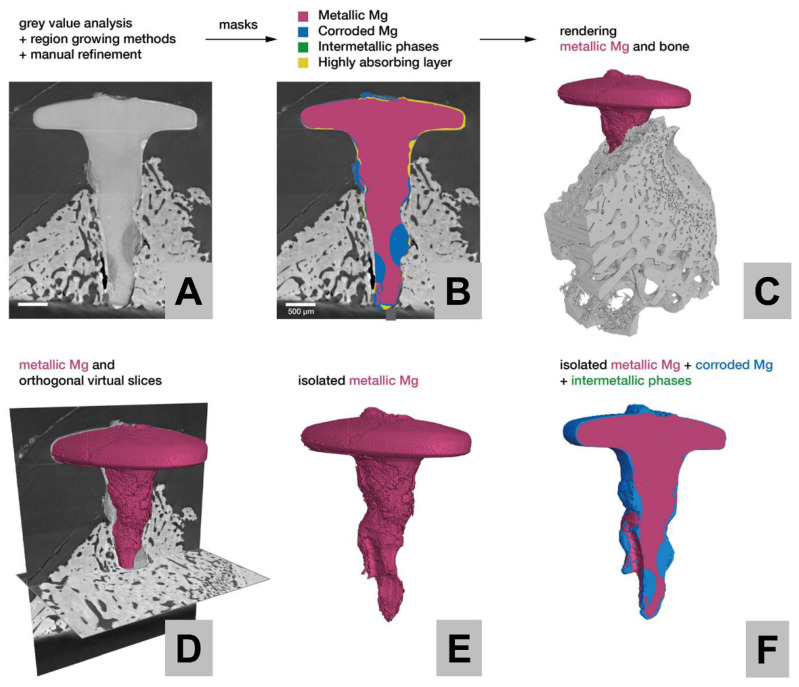
(**A**) Virtual section through the reconstructed volume of an HF-treated Mg alloy screw positioned within the bone tissue at 3 weeks post implantationem. (**B**) Segmentation of the screw into its different components, namely metallic Mg, Mg salts, intermetallic particles and a fast absorbing corrosion front. (**C**) Rendering of the metallic Mg screw together with the surrounding bone tissue. (**D**) Rendering of the metallic Mg together with two orthogonal virtual sections. (**E**) Remaining bulk Mg metal structure of the screw has been isolated from the surrounding bone and corrosion products, whereas in (**F**), the Mg screw has been rendered to depict the bulk Mg metal structure as well as the remaining solid Mg corrosion byproducts with a virtual cut through the center of the screw.

Additionally, the biopsies were also scanned using synchrotron-based phase contrast micro-CT to analyze the corrosion behavior of the Mg alloy screws (Figure 2). Due to a higher image quality (smaller voxel size) compared to standard laboratory micro-CT, the synchrotron-based phase contrast micro-CT scan is capable of characterizing the distribution of corrosion over the Mg screws in 3D.

**Figure 3:**
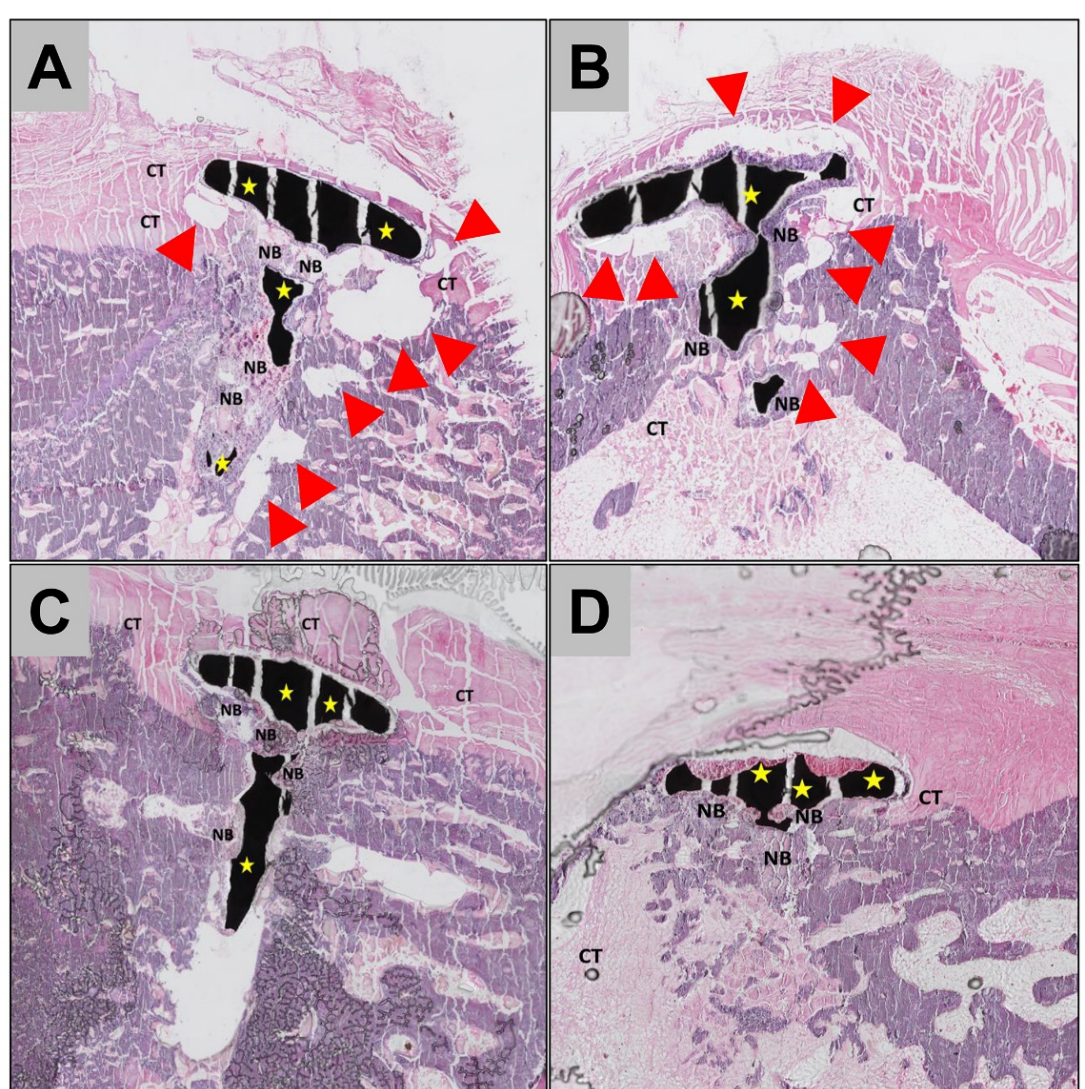
Representative histological images of the integration behavior and the tissue reactions to the (**A**,**B**) untreated Mg screws and (**C**,**D**) the HF-treated Mg screws (yellow stars) after 3 weeks (left column) and 6 weeks post implantationem (right column). Bot Mg screw types were widely integrated into newly formed bone tissue (NB), but in the group of the untreated screws high numbers of gas cavities (red arrows in **A**,**B**) were detected within the surround bone and connective tissue (CT). Please note that the missing part of the Mg screw in B does not reflect the overall level of biodegradation (“total scans”, HE—stainings, 100× magnifications).

The data reveal the presence of a metallic core for each of the screws, along with a fast-absorbing corrosion front followed by a slower absorbing region of corroded Mg in the form of Mg salts (Figure 3). The 3D image analysis clearly reveals that the corrosion of the Mg alloy screw progresses from the surface to the inside. The most pronounced corrosion can be seen along the shaft of the screw.

### 2.3. Histopathological Results

**Figure 4:**
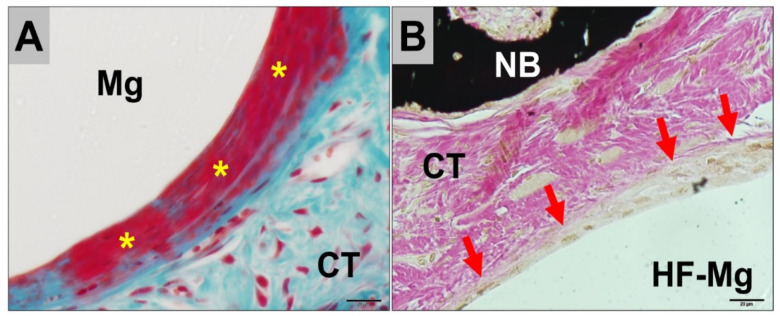
Representative histological images of the tissue responses to the analyzed biomaterials at 3 weeks post implantationem. (**A**) The untreated Mg screws (Mg) induced a low-grade fibrous encapsulation (yellow stars). CT, connective tissue (Masson Goldner staining, 40× magnification). (**B**) In contrast, the HF-treated screws (HF-Mg) showed a phagocyte accumulation at their surfaces (red arrows) (CT, connective tissue, NB, newly formed bone matrix) (von Kossa staining, 40× magnification, scalebar = 20 µm).

In all study groups, the Mg screws were found within their implantation beds at 3- and 6 weeks post implantationem (Figure 3 and Figure 4). The histopathological analysis showed that the Mg screws were mainly integrated within bone tissue in all study groups and at both study time points (Figure 3). Moreover, for both screw types, in the areas where the Mg screws had completely corroded, the screws were replaced by newly formed bone (Figure 3 and Figure 4).

**Figure 5:**
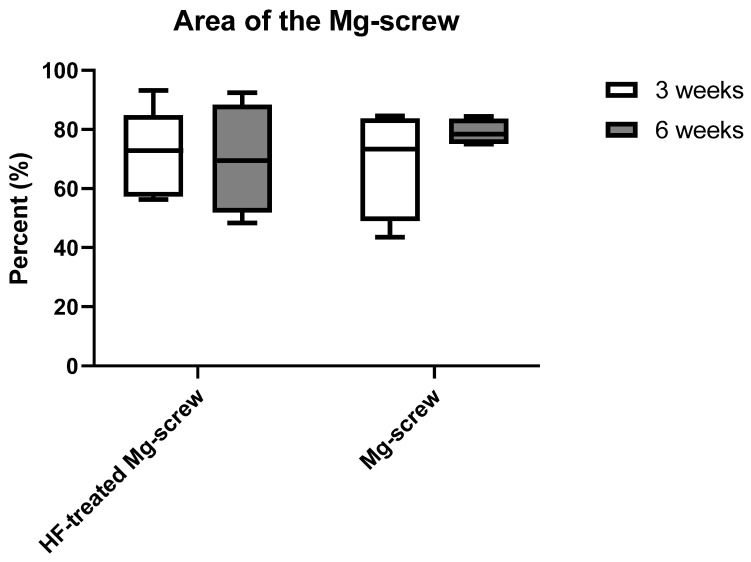
Results of the histomorphometrical measurements of Mg screw areas.

In the areas in which the Mg screws were neighbored to connective tissue, a different tissue response was observed for the untreated and the HF-treated screws (Figure 5). In the group of the untreated screws, a low-grade fibrous encapsulation was detected at the implant–tissue interface, as well as gas cavities within the surrounding connective tissue at both study time points (Figure 4A). However, no signs of adverse tissue reactions or compromised (bone) tissue healing have been detected caused by the gas cavities (Figure 3).

In contrast, a layer mainly consisting of macrophages was observed at the implant–tissue interface in the groups of the HF-treated Mg screws up to 6 weeks post implantationem (Figure 4B). In this study group, gas cavities were either not present or were fewer in comparison to the groups of the untreated Mg screws (Figure 4). There were no signs of an exaggerated inflammatory tissue response to the Mg screws observed for either study group.

### 2.4. Histomorphometrical Results

#### 2.4.1. Measurement of the Remaining Mg screw Areas

The histomorphometrical analysis showed that comparable areas of the remaining Mg alloy screw were measured in all study groups without any statistical differences between study time points (Figure 5).

#### 2.4.2. Measurements of the Mg screw Diameters

**Figure 6:**
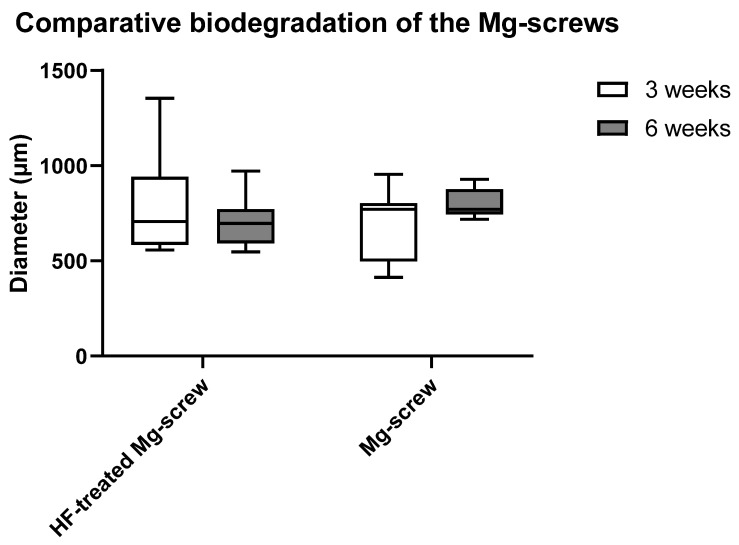
Results of the histomorphometrical analysis of the biodegradation of the Mg-based screws via diameter measurements.

The histomorphometrical analysis for the biodegradation of both types of Mg alloy screws via diameter measurements showed that no significant differences were present between all study groups (Figure 6).

#### 2.4.3. Measurements of the Implant-Bone-Contact

**Figure 7:**
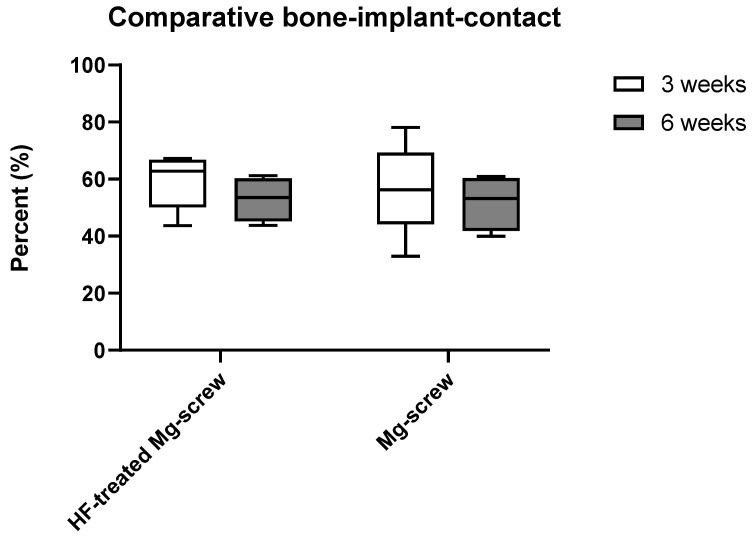
Results of the histomorphometrical measurements of the implant-bone-contact in all study groups.

The measurements of the implant–bone contact of the two Mg-based screw types showed no significant differences between the study groups (Figure 7).

#### 2.4.4. Material-Related Gas Cavity Measurements

**Figure 8:**
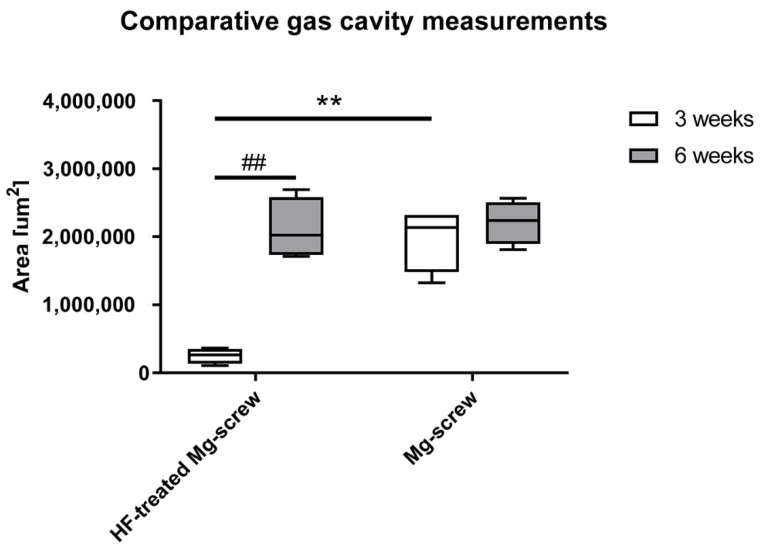
Results of the histomorphometrical measurements of gas cavity sizes in all study groups. Significant differences are marked with asterisks (##/** *p* ≤ 0.01).

A comparison of material-related gas cavity sizes indicated that the size of gas cavities produced during the corrosion of the HF-treated Mg screws were significantly lower compared to the untreated Mg screws (** *p* ≤ 0.01) (Figure 8).

#### 2.4.5. Element Analysis of the Implantation Beds

**Figure 9:**
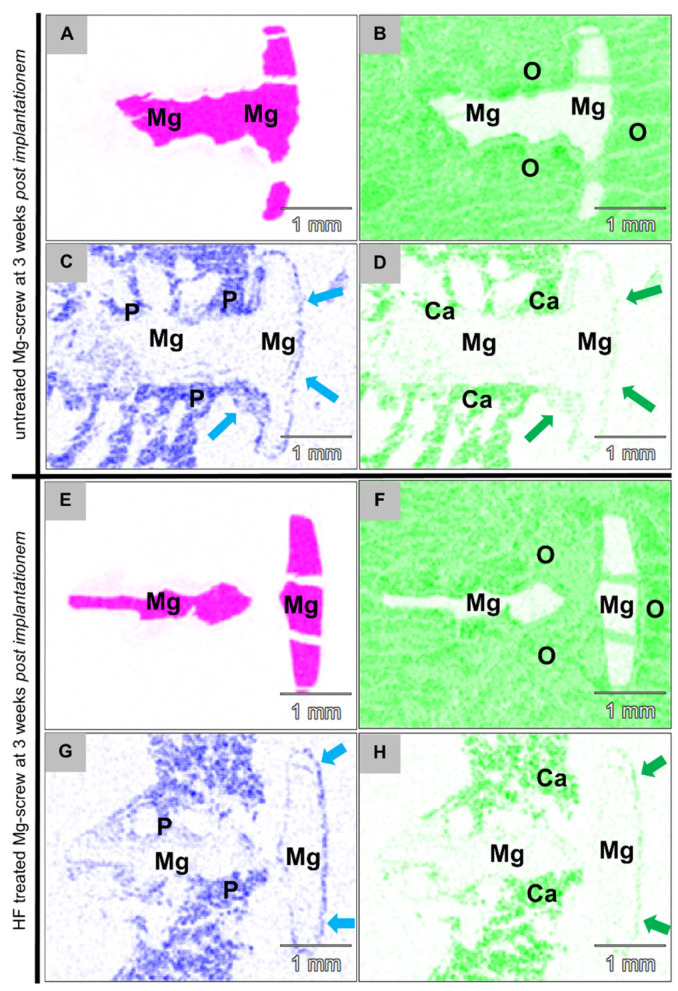
EDX distribution pattern from the implantation bed of an (**A**–**D**) untreated Mg screw (Mg colored pink) and (**E**–**H**) HF-treated Mg screws at 3 weeks post implantationem. The images show the detection of phosphate ions (P colored blue, left column) within the trabecular bone surrounding the screw shaft. Moreover, calcium ions (Ca colored green, right column) were detectable. The phosphate and calcium ions at the interface of the screw heads (blue and green arrows) located within the neighbored connective tissue indicate the presence of magnesium salts formed as part of the corrosion process (70× magnification, scalebars = 1 mm).

**Figure 10:**
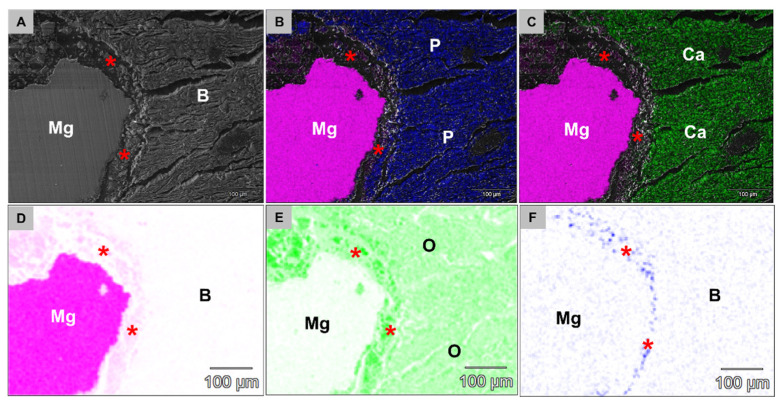
SEM/EDX images from the tissue interface of the HF-treated Mg screw heads at 3 weeks post implantationem. (**A**) The SEM imaging showed that the screw heads were integrated within newly formed bone tissue (**B**) that was composed of (B) phosphate (P) and (**C**) calcium ions (Ca). An additional layer (red asterisks) was detectable at the material surfaces that was composed of (**D**) magnesium ions, oxygen corrosion species (**E**,**F**) fluoride indicating the MgF_2_ layer (500× magnification, scalebars = 100 µm).

The combined analysis via scanning electron microscopy (SEM) and energy dispersive X-ray spectroscopy (EDX) revealed that both Mg alloy screw types were integrated within bone tissue at 3 and 6 weeks post implantationem (Figure 9). A surface composed of phosphate and calcium ions was detected on the screws, even in the position of the screwheads that were located within connective tissue at both study time points (Figure 9). At 3 weeks post implantationem, a layer composed of fluoride and magnesium ions observed at the surfaces of the HF-passivated Mg screws (Figure 10).

**Figure 11:**
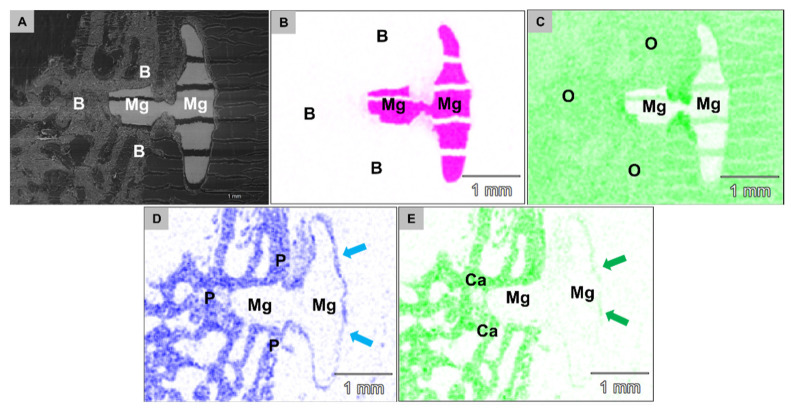
SEM/EDX images from the implantation bed of an HF-treated Mg screw at 6 weeks *post implantationem*. (**A**) The SEM imaging showed that the Mg screw shafts were still integrated within newly formed bone tissue. Detection of (**B**) the magnesium implant (Mg) surrounded by an (**C**) oxygen (O)-rich tissue and (**D**) phosphate (P) and (**E**) calcium ions (Ca). An additional layer (blue and green arrows) of phosphate and calcium was still detectable at the screw head surfaces (70× magnification, scalebars = 1 mm).

**Figure 12:**
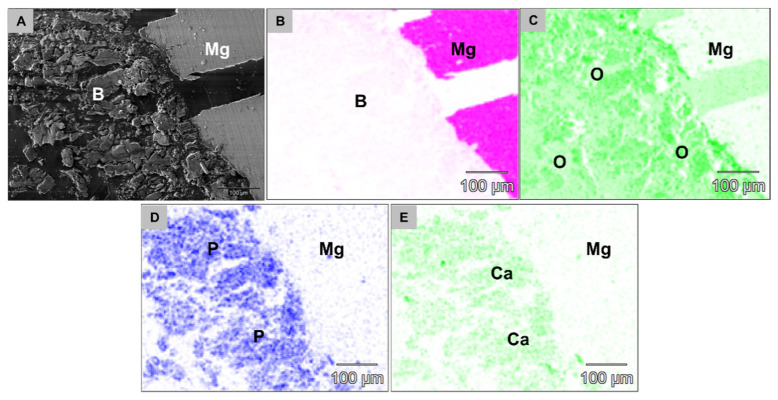
SEM/EDX images from tissue interface of an HF-treated Mg-based screw at 6 weeks *post implantationem*. (**A**) The SEM imaging show that the screws were still integrated within newly formed bone tissue (**B**). No magnesium ions (B) but (**C**) oxygen, (**D**) phosphate (P) and (**E**) calcium ions (Ca) were detected within the surrounding tissue indicating the absence of the previously detected layer (500× magnification, scalebars = 100 µm).

At 6 weeks post implantationem, the analysis showed that the Mg screw shafts were still integrated within newly formed bone tissue and the layer of phosphate and calcium was still detectable at the screw head surfaces (Figure 11). Thereby, the aforementioned layer composed of fluoride and magnesium ions was not detectable at 6 weeks post implantationem in the group of the HF-treated Mg screws (Figure 12).

**Figure 13:**
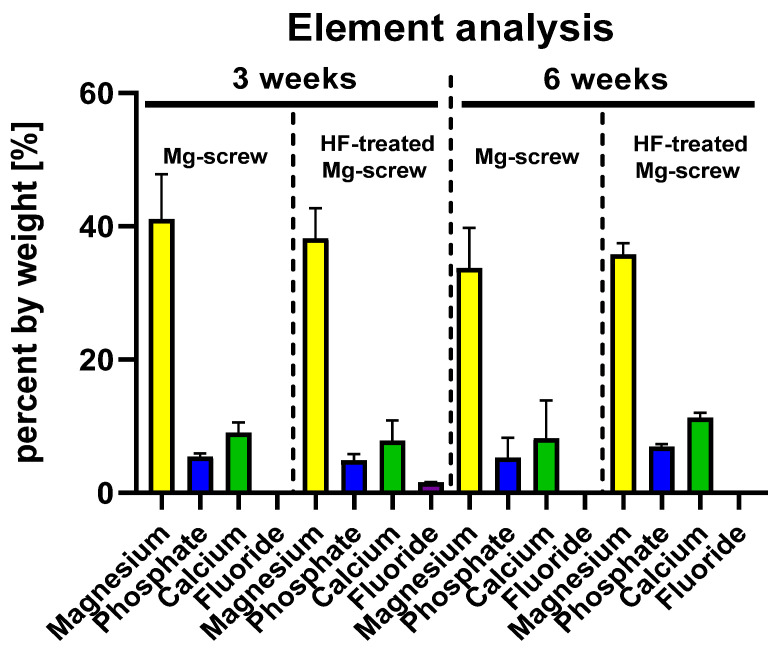
Element analysis based on SEM/EDX detection within the implantation beds of the Mg alloy screws at 3 and 6 weeks post implantationem.

The quantitative analysis of the element distribution within the implantation beds of both Mg screw types revealed that magnesium was prevalent within the implantation beds to a comparable extent (~40%) for all specimens at 3 and 6 weeks post implantationem (Figure 13). Furthermore, comparable extents of phosphate (~6%) and calcium (~10%) were measured within the implantation beds of all specimens at both time points (Figure 13). Only in the group of the HF-passivated Mg screws was a low fluoride presence (1.6%) detected at 3 weeks post implantationem (Figure 13).

## 3. Discussion

GBR therapy is based on the use of barrier membranes to enable regeneration of lost bone tissue in dentistry, maxillofacial surgery and other surgical disciplines [9,15,48,49,51,52]. To stabilize these membranes and hence the underlying bone substitute during the healing process, the use of fixation screws has been proven to be an effective approach [53,54,55]. Non-resorbable fixation systems made of titanium (Ti) are the most widely used applications for this purpose, due to their good mechanical properties and biocompatibility, but are also associated with typical disadvantages, similar to delayed osteointegration and microbial colonization [56,57]. An additional concern is the reported accumulation of material particles around Ti and ceramic implants [58,59] However, the main disadvantage for non-resorbable fixation screws is the requirement for their subsequent surgical removal and the resulting risks for the patient [9,49,56,57,60]. Bioresorbable screws, made from polymers such as polylactide and polyglycolide and their copolymers are also available [22,23,61,62]. The good biocompatibility of this material class has been proven several times, but they show occasionally unstable degradation processes and an acidic degradation profile, which can inhibit tissue regeneration [26,27,28].

Magnesium (Mg) is a bioresorbable metal that releases magnesium ions as it degrades that are naturally prevalent within the human body [15,16,17,18]. Due to its mechanical and biocompatible properties, Mg represents a promising alternative to common materials used for fixation screws [9,15,16,17,18]. However, the degradation of magnesium in aqueous environments results in the release of hydrogen gas, which if uncontrolled, can interfere with the regeneration processes [9,15,16,17,18]. Passivation of the magnesium materials presents a promising option that can benefit biocompatibility, regenerative effects and mechanical properties [15,16,17]. To prolong the degradation time and thus reduce the hydrogen release, the surface of the magnesium implant can be treated with hydrofluoric acid. This surface passivation technique has already been used in various clinical applications [63,64,65]. By treating the surface with hydrofluoric acid, a magnesium fluoride (MgF_2_) layer is produced, which has previously been demonstrated to improve corrosion resistance [15,66].

The aim of the present study was to analyze the cyto- and biocompatibility, the biodegradation behavior and the bony integration of newly developed Mg alloy screws treated with or without HF. Initially, an in vitro analysis based on ISO 10993-5/-12 that had been adapted for Mg-biomaterials was performed that included viability (XTT), proliferation (BrdU) and cytotoxicity (LDH) assays [16,17]. This was followed by an in vivo study, where the screws were implanted into the distal femur of 40 New Zealand rabbits for 3 and 6 weeks. The in vivo analysis included established histological work-up methods as well as previously published histopathological and histomorphometrical procedures [15,45,46,47,48,49,50]. Element analysis based on scanning electron microscopy (SEM) combined with energy dispersive X-ray spectroscopy (EDX) was performed on biopsies, with a special focus on the quantitative and qualitative detection and element distribution within the implantation beds of the screws.

In vitro, clear cytocompatibility was demonstrated for the HF-treated Mg alloy screws. The statistically minor (XTT) and strong (BrdU) differences compared with the titanium grade 5 negative control suggest that minor proliferation and viability inhibition occurred, which were negligible. The untreated screws, however, showed clear and statistically highly significant differences in comparison with the negative control assuming lower cytocompatibility. However, this result can be explained by the in vitro conditions, which do not replicate those in vivo. The ISOs recommended static media conditions and low extraction volume lead to extensive changes in pH and osmolality for magnesium materials that lead to cytotoxic results for an otherwise biocompatible material, and is a well-recognized occurrence [16,67].

In the LDH assay, the values of the HF-treated screws were significantly below the cytotoxicity limit and differed strongly and significantly from the positive control. The untreated Mg screws exceeded the threshold. These results correspond with our previous in vitro investigations with untreated magnesium materials [16,17]. Altogether, the results of this study part indicate sufficient cytocompatibility and improved degradation behavior of the HF-treated screws.

The results of the cytocompatibility assays coincide with earlier investigations on the degradation-retarding properties of HF-treated magnesium-based materials [43,68,69]. In a previous study, Jung et al. were able to show similar positive effects on cell vitality and proliferation for the in vitro cytocompatibility analysis of an HF-treated Mg membrane for guided bone regeneration (GBR) [15]. Therefore, the positive effects on cytocompatibility of the investigated Mg biomaterials may be directly attributed to the protective properties of the HF-treatment by providing a controlled degradation with a delayed onset. This prevents (a) the rapid release of hydrogen gas bubbles, which may have a harmful effect on the surrounding cells, (b) the accumulation of hydroxide ions and thus alkalization of the surrounding medium, which may negatively affect cell growth, and (c) the creation of a hyperosmolar solution which may negatively affect cell proliferation, as demonstrated by the BrdU assay for the untreated screw [68,69,70,71].

Histopathological analysis of the untreated and the HF-treated Mg alloy screws implanted in vivo, revealed that each screw type induced a different tissue reaction. The untreated Mg screws induced a tissue response that included a low-grade fibrous encapsulation and frequent gas cavity formation within the surrounding tissues. In contrast, the tissue reaction to the HF-treated screws included a phagocyte accumulation at the screw surfaces and infrequent signs of gas cavity formation up to 6 weeks post implantationem. These results are consistent with the observations of a previous in vivo study which compared HF-treated and untreated Mg alloy barrier membranes [15]. Results from the present study suggest a similar tissue reaction pattern of the Mg screws.

The HF surface passivation for the Mg screws seems to induce phagocytosis and the accumulation of phagocytes. This can be seen as a sign of a low foreign body reaction [15]. Interestingly, a similar tissue response was shown for the previously reported HF-treated membranes after a period of 18 weeks [15]. At which point, the MgF_2_ surface appears to be largely degraded and the untreated magnesium encounters the surrounding tissue. In contrast, the SEM/EDX analysis of the Mg screws only detected an MgF_2_-layer at 3 weeks post implantationem but not after 6 weeks. However, the degradation behavior and gas cavity formation were also decreased at both time points, which is in accordance with the delayed corrosion behavior reported for the magnesium meshes, as well as in various other studies investigating MgF_2_-based coatings [68,69,72].

Micro-CT-based measurements further support the benefit of HF surface passivation of the Mg screws, as at both time points there was significantly more of the material volume remaining. However, delayed onset of Mg degradation was not supported by the histomorphometrical measurements on the remaining screw area and diameter. Other studies have also reported on a lack of correlation between micro-CT-based 3D-measurements and histological assessment, while 2D micro-CT data and histomorphometrical measurements have shown to lead to comparable results [73,74]. It has also been previously reported that histomorphometrical data based on histological slides could allow for a more exact evaluation for areas of specific evaluation, such as (early) bone regeneration [75].

Synchrotron-based phase contrast micro-CT analysis of Mg screw corrosion revealed that there was a fast absorbing corrosion front as well as a slowly absorbing region composed of the corroded magnesium metal. It also demonstrated that the corrosion of the Mg alloy screw acted on the surface of the implant.

With the addition of SEM/EDX analysis, it was concluded that the corrosion front was composed of calcium and phosphate, most likely as an oxide layer [76,77]. The presence of these ions might lead to a conversion that may be beneficial for the bone regeneration process, as the shafts of both screw types were optimally integrated within bone matrix. The screw heads that were located within the soft tissue were covered by a calcium phosphate-containing layer.

For future analysis of the exact degradation and conversion behavior of Mg alloy biomaterials, a more detailed synchrotron-based micro-CT examination with phase contrast should be used due to its superior contrast for small variations of the local mass density to provide deeper insights into the molecular mechanisms.

Altogether, it can be assumed that both screw types differ in the quality of degradation, since the MgF_2_-layer initially decreases the corrosion rate of the HF-treated screws. Nevertheless, these results only represent an in vivo behavior of the passivated screws in animal models, and to obtain meaningful results with regard to therapeutic use in humans, appropriate studies for clinical validation will be required in the future.

The current study analyzed the biocompatibility and degradation behavior of the HF-passivated screws; however, it did not evaluate their mechanical properties nor their stability under mechanical load or stress yield. Additional biomechanical testing of the Mg alloy screws is required in order to be able to make concrete statements about their overall clinical functionality.

Altogether, the present results have demonstrated that both Mg screw types did not induce undesirable tissue reactions and were integrated within bone tissue to comparable levels. However, the HF-treated screws did induce significantly fewer gas cavities and a significantly reduced corrosion rate. Moreover, these results lead to the conclusion that the HF-treatment of the Mg screws has been effective in reducing the corrosion rate compared to untreated Mg screws. Thus, the HF-treated screws seem to be more suitable for the intended clinical indication, as this medical shows an overall better biocompatibility compared to the untreated screws.

Based on these results, it can be assumed that the tested biomaterials can be applied clinically without causing side effects. The present results clearly demonstrates that the analysis of newly developed complex biomaterials, such as the analyzed HF-treated screws, require a combined analysis involving different methods. Only a combination of results including histopathology and histomorphometry, as well as SEM/EDX analysis enabled an overall impression of the degradation behavior and biocompatibility of the newly developed medical device. This novel resorbable biomaterial—even in combination with resorbable materials such as collagen membranes or bone substitutes—can change the GBR concept and finally minimize the number of required surgical interventions as fixation materials. The present study furthermore includes novel data even in view of the degradability analysis of Mg-based biomaterials and coating technologies based on a combined SEM/EDX analysis and synchrotron-based phase contrast micro-CT.

## 4. Materials and Methods

### 4.1. Biomaterial Preparation

The NovaMag^®^ fixation screw (botiss biomaterials GmbH, Zossen, Germany) and a fixation screw with equal dimensions to the NOVAMag^®^ fixation screw, but without an HF-treated surface (botiss biomaterials GmbH, Zossen, Germany), were used in this study. The screws had a size of Ø 1.0 × 3.5 mm. All samples were ultrasonically cleaned in 100% ethanol and distilled water. For extraction, 2 screws per well were added to the extraction medium. All materials were sterilized via gamma irradiation.

### 4.2. Cytocompatibility Analysis

Cytocompatibility tests were performed following ISO 10993-5/12 and described in detail in previous publications [16,17,78]. The experimental setup is therefore summarized in brief.

#### 4.2.1. Reference Material

Reference materials were immersed in isopropanol for 5 min and then dried in a constant laminar flow. RM-A ZDEC Polyurethane Film (Hatano Research Institute, Food and Drug Safety Center, Ochiai, Japan) was used as positive control. Titanium grade 4 and grade 5 was used as negative control material. RM-A specimens and titanium plates were utilized with the same surface areas as the test materials and sterilized in the same way.

#### 4.2.2. Cells and Cell Culture

For the assays, L-929 mouse fibroblasts and mouse osteoblast precursor cells MC3T3 were purchased from the European Collection of Cell Cultures, ECACC (Salisbury, UK). Cultivation was performed under cell culture conditions (37 °C, 5% CO_2_ and 95% humidity) in MEM (Minimum Essential Medium, further referred as cell culture medium) supplemented with 10% FBS (fetal bovine serum), penicillin/streptomycin (100 U/mL each) (all from Life Technologies, Carlsbad, CA, USA) and L-glutamine (Sigma-Aldrich, St. Louis, MO, USA) to a final concentration of 4 mM. Passage was performed at a confluence of 80%. Thereby, 4 screws per material type were sued for every test parameter in vitro.

#### 4.2.3. Extract Analysis

##### Extraction

Both test and control samples were extracted for 72 h in cell culture medium and under cell culture conditions as described above. According to DIN EN ISO 10993-12, the surface to volume ratio for the test samples was 3 cm^2^/mL. Cell culture medium alone was cultured as a blank sample under the same conditions. After extraction, the test samples were removed, and the extracts were centrifuged at 14,000 rpm for 10 min.

##### Assay Procedure

Next, 96-well plates were grown with 1 × 10^4^ cells/well in 100 µL cell culture medium and cultured for 24 h under cell culture conditions. Afterward, the cell culture medium was removed and 100 µL/well of the extract solutions were added. After another 24 h incubation, the cells were analyzed utilizing BrdU- and XTT-assays. The supernatants were analyzed by LDH assay. To measure possible interferences, 100 µL of the pure extracts (without cells) were also subjected to identical assays. Measured absorbance values of the blank samples were subtracted from all results of the extract assays.

##### Bromodeoxyuridine/5-Bromo-2′-Deoxyuridine (BrdU)-Assay

A BrdU (colorimetric) test kit (Roche Diagnostics, Mannheim, Germany) was used for testing. According to manufacturer’s instructions, cells were incubated for 2 h with BrdU under cell culture conditions and fixed afterward with FixDenat reagent for 30 min at room temperature. Cells were then incubated for 1 h with an anti-BrdU peroxidase (POD) antibody and subsequently rinsed 3 times for 5 min each with washing buffer. Tetramethyl-benzidine (TMB) was added to the substrate and the resulting reaction was stopped after 20 min under room temperature by adding 25 µL of 1 M H_2_SO_4_. Finally, the formed immune complexes were measured by a scanning multi-well spectrophotometer (ELISA reader) at wavelengths of 450 and 690 nm (reference wavelength).

##### Sodium 3,3′-[1(Phenylamino)carbonyl]-3,4-tetrazolium]-3is(4-methoxy-6-nitro) Benzene Sulfonic Acid Hydrate (XTT)-Assay

A Cell Proliferation Kit II (Roche Diagnostics, Mannheim, Germany) was used for testing. According to the manufacturer’s instructions, the electron-coupling reagent was mixed with the XTT labeling reagent (1:50 dilution) and 50 µL of the resulting solution was added to the cells. After 4 h of incubation under cell culture conditions, 100 µL aliquots were transferred to a new 96 well plate and the substrate conversion was measured via scanning multi-well spectrophotometer (ELISA reader) at wavelengths of 450 and 650 nm (reference wavelength).

##### Lactate Dehydrogenase (LDH)-Assay

An LDH Cytotoxicity Assay Kit II (BioVision, Milpitas, CA, USA) was used for testing. According to the manufacturer’s instructions, 10 µL of cell supernatants were incubated with 100 µL LDH reaction mix for 30 min at room temperature. Stop solution was then added and absorbance was measured via scanning multi-well spectrophotometer (ELISA reader) at wavelengths of 450 and 650 nm (reference wavelength).

### 4.3. Pre- and Post-Implantation Procedure, Surgical Procedure

The in vivo study was approved by the Local Ethical Committee and Ministry of Agriculture, Forestry and Water Management of the Republic of Serbia (Faculty of Medicine, University of Niš, Serbia, No. 323-07-00278/2017-05/4, date: 10 June 2017). Animals were obtained prior from the Military Medical Academy (Belgrade, Serbia) and randomly assigned to the different study groups, i.e., (1) HF-treated Mg screw and (2) untreated Mg screw and implanted and two time points (3 and 6 weeks). In total 20 animals were used for this study with n = 5 rabbits per study group and timepoint. Before and after implantation, the experimental animals were kept under standard conditions with regular pellets, access to water ad libitum and an artificial light–dark cycle of 12 h each.

**Figure 14:**
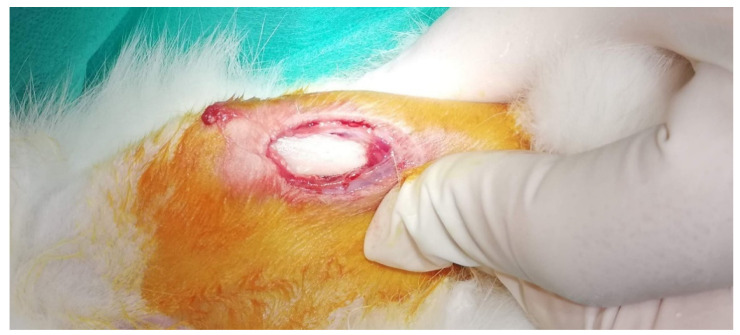
Implantation site showing the exposed tibia.

After anesthesia by an intraperitoneal injection of 10 % pentobarbital (30 mg/kg body weight) (Dainippon Sumitomo Pharma, Osaka, Japan), the surgical field was prepared, shaved and disinfected. Afterward, a small skin incision over the bony implantation site was made using a scalpel, and the underlying soft tissue as well as the periosteum down to the medial tibia was frankly prepared using surgical forceps (Figure 14).

Then, a surgical drill with a drill head of 1 mm in diameter (Ustomed, Tuttlingen, Germany) was used for pre-drilling. Immediately after this procedure, the respective Mg screw was implanted. Finally, the soft tissue was closed over the implantation site and the skin was removed and sutured via resorbable suture material (Maricryl 4/0, Markneukirchen, Germany).

After the healing periods of 3 and 6 weeks, the explantation and the histological workup were conducted as previously described [45,79,80,81]. In brief, the implanted Mg screws were cut out together with the peri-implant bone and soft tissue immediately after the euthanasia. Afterward, the explants were fixed in 4% neutral-buffered formalin for 2 days.

### 4.4. Synchrotron Micro-CT

Exemplarily, one PMMA-embedded screw has been CT scanned at the Anatomix beamline of the Synchrotron SOLEIL [82,83]. The pink-beam central energy was 40 keV. The effective detector pixel size was 3.07 µm. Acquisition time per projection was 50 ms. The rotation axis was set off the center to increase the final reconstructed volume to 3748 voxels in diameter and 2048 voxels in height, with a voxel size identical to the effective detector pixel size. A total of 5600 projections were collected, resulting in a total scanning time of less than 7 min, including collection of flat and dark field images. Reconstruction was performed using the PyHST2 software [84] using a Paganin filter in combination with the conventional filtered back projection algorithm applying a Paganin length of 50 pixels. Image segmentation and visualization was performed using the segmentation tools of the AVIZO software (Avizo 9.7.0, Thermo Fischer Scientific Inc., Waltham, MA, USA).

### 4.5. Histological Workup

Further histological workup was performed following the standard Technovit 9100 new protocol. In brief, all explants were subsequently embedded in plastic and sections were cut with a thickness of 3–5 µm using a rotation microtome (Slee cut 6062, SLEE medical, Mainz, Germany). Histochemical staining was also performed as previously described [45,79,80,81]. Slides of the tissue blocks from each animal were stained with hematoxylin and eosin (H&E) for histopathological evaluation of the tissue reactions to the Mg screws and histomorphometrical analysis of their bony integration.

### 4.6. Histopathological Analysis

The qualitative histopathological analysis was conducted following a previously published protocol [45,79,80,81]. In brief, parameters such as (inflammatory) tissue reaction to the implants, cells participating in the process of Mg screw integration and degradation, implantation bed vascularization and possible adverse reactions such as fibrotic encapsulation or necrosis were analyzed. A light microscope (GmbH, Axio Imager A2, Carl Zeiss Microscopy GmbH, Deutschland) was used for the analysis. Histological figures were taken by a microscope camera (Axiocam 506 color, Carl Zeiss Microscopy GmbH, Deutschland).

### 4.7. Histomorphometrical Analysis

The histomorphometrical analysis was conducted based on previously published and established methods [45,79,80,81]. The analysis included the comparative measurements of the remaining area of the Mg screws, i.e., the biodegradation, and the bone-implant-contact as well as the gas cavity measurements.

Briefly, so-called “total scans” were generated with the aid of a specialized scanning microscope, which consisted of an Eclipse 80i histological microscope combined with a DS-Fi1 digital camera and an automatic scanning table (Prior Scientific, Rockland, MA) connected to an PC system running the Zeiss software (Carl Zeiss Microscopy GmbH, Deutschland). The resulting images were composed of 100 to 120 single images with a 100x magnification in a resolution of 2500 × 1200 pixels and contained the complete implant area as well as the peri-implant tissue of the Mg screws.

To calculate the biodegradation of the Mg screws, both the remaining areas of the Mg screws in both study groups were measured. Furthermore, the diameters in three parts of the screws were measured, divided by the respective ex vivo diameters of the different areas of the Mg screws, and finally presented as a percentage.

Furthermore, the bone–implant contact of the screws, i.e., the insertion area, was histomorphometrically analyzed. Using the length measurement tool of the Zeiss software (Carl Zeiss Microscopy GmbH, Deutschland), the area of the screw in direct contact with the bone was compared to its initial insertion area and presented as a percentage.

The area measurement tool of the Zeiss software (Carl Zeiss Microscopy GmbH, Deutschland) was used to determine the area of gas cavities within the peri-implant tissue of the Mg screws of all groups.

### 4.8. Elemental Analysis within the Implantation Beds

The morphology and element distribution of the transection were characterized by SEM/EDX using a LEO Gemini 1530 with a field-emission gun from Carl Zeiss AG (Jena, Germany). Samples were precoated with carbon prior to scanning electron microscopy (SEM) and energy-dispersive X-ray spectroscopy (EDX) to prevent the effect of charging on the samples. The EDX maps (256 × 196 pixels) were acquired with a Thermo Noran X-ray detector and the Thermo Fisher Scientific software Noran System Six. The applied voltage was set to 5 kV for imaging and 10 kV for EDX mapping.

### 4.9. Statistical Analysis

The data were statistically analyzed by an analysis of variance (ANOVA) followed by LSD post-hoc assessment to compare groups using SPSS 16.0.1 software (SPSS Inc., Chicago, IL, USA). The differences were considered significant if *p* values were less than 0.05 (* *p* ≤ 0.05), and highly significant if *p* values less than 0.01 (** *p* ≤ 0.01) or less than 0.001 (*** *p* ≤ 0.001). The data were presented as mean ± standard deviation using the GraphPad Prism 6.0c software (GraphPad Software Inc., La Jolla, San Diego, CA, USA).

## Figures and Tables

**Table 1:** Results of the different cytocompatibility assays. Values given in %. SD, standard deviation.

Assay	Medium Control	Titanium Grade 4	Titanium Grade 5	Positive Control	HF-Treated Screw	Untreated Screw
Proliferation (BrdU)	100 ± 1.64	96.06 ± 1.66	96.4 ± 3.04	1.55 ± 0.53	90.32 ± 2.13	0.06 ± 0.18
Viability (XTT)	100 ± 1.12	100.37 ± 3.55	100.13 ± 2.05	−0.51 ± 0.12	89.79 ± 0.64	1.70 ± 0.23
Toxicity (LDH)	100 ± 43.0	66.8 ± 11.9	58.7 ± 39.2	3886.4 ± 169.7	−7.6 ± 36.8	2832.0 ± 97.3

## Data Availability

Data is contained within the article.
